# Sex-specific utility of pulmonary artery metrics in predicting pulmonary hypertension and survival after TAVI: insights from advanced CT imaging

**DOI:** 10.1186/s13244-026-02272-x

**Published:** 2026-04-07

**Authors:** Elke Boxhammer, Matthias Hammerer, Nikolaos Schörghofer, Nikolaus Clodi, Erika Prinz, Wilfried Wintersteller, Uta C. Hoppe, Klaus Hergan, Christoph Knapitsch, Bernhard Scharinger

**Affiliations:** 1 Department of Internal Medicine II, Division of Cardiology, Paracelsus Medical University of Salzburg, Salzburg, Austria; 2 Department of Radiology, Paracelsus Medical University of Salzburg, Salzburg, Austria

**Keywords:** TAVI, Computed tomography, Main pulmonary artery, Pulmonary hypertension, Sex-specific difference

## Abstract

**Objective:**

Pulmonary hypertension (PH) significantly affects outcomes after transcatheter aortic valve implantation (TAVI), with sex-specific differences indicating the need for tailored strategies. This study investigated the predictive value of CT-derived main pulmonary artery (MPA) dimensions and ratios, focusing on diagnostic accuracy and prognostic relevance in male and female TAVI patients.

**Materials and methods:**

A retrospective analysis of 526 patients (263 male, 263 female) undergoing TAVI was performed. PH was defined echocardiographically according to European Society of Cardiology (ESC) guidelines. Pre-procedural CT measurements of MPA, ascending aorta (AA), and derived ratios (e.g., MPA/AA) were analyzed. Sex-specific cut-offs were determined using area under the receiver operating characteristic (AUROC) analyses and validated with survival curves and Cox regression.

**Results:**

MPA and its ratios outperformed right and left pulmonary artery metrics in detecting PH. Overall cut-offs were MPA ≥ 29.5 mm and MPA/AA ≥ 0.76. In men, elevated MPA or MPA/AA showed strong associations with PH, whereas in women, higher cut-offs (MPA ≥ 30.0 mm; MPA/AA ≥ 0.86) were less diagnostically useful. Importantly, the MPA/AA ratio predicted long-term survival only in men (hazard ratio (HR) = 1.857, *p* = 0.006), underlining its limited prognostic role in females.

**Conclusion:**

CT-derived pulmonary artery metrics are valuable for predicting PH and survival in male TAVI patients. Incorporating the MPA/AA ratio into clinical practice may improve risk stratification in men, while limited diagnostic utility in women highlights the need for alternative markers. Sex-specific approaches should be pursued to optimize outcomes across all PH etiologies.

**Critical relevance statement:**

CT-derived pulmonary artery metrics reliably predict PH and long-term survival after TAVI, particularly in men, emphasizing their diagnostic and prognostic value while underscoring the need for sex-specific thresholds and alternative markers in women.

**Key Points:**

- PH impacts TAVI outcomes, yet sex-specific radiological predictors remain insufficiently investigated.
- The pulmonary artery to AA ratio predicted survival in men but showed no prognostic value for women.
- Implementing sex-specific imaging assessments improves risk stratification in men, highlighting the need for distinct diagnostic strategies for women.

**Graphical Abstract:**

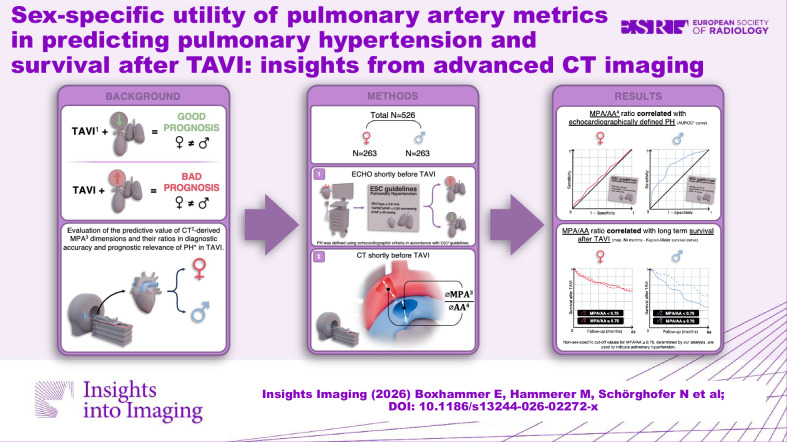

## Introduction

Transcatheter aortic valve implantation (TAVI) has become a cornerstone in the management of severe aortic stenosis (AS), particularly for patients considered high-risk for traditional surgery [1]. However, the presence of pulmonary hypertension (PH) in TAVI candidates complicates long-term survival [2, 3], and its impact on outcomes has become an increasing area of interest. The relationship between PH and TAVI outcomes may differ based on sex [4, 5], highlighting the need to consider biological differences in clinical decision-making. Despite advances in TAVI techniques, the full impact of PH, especially in the context of AS, remains underexplored regarding its influence on post-procedural prognosis.

Traditionally, PH in TAVI candidates has been assessed using echocardiography, which estimates pulmonary pressures and evaluates right heart function [3, 6, 7]. However, echocardiography alone often fails to provide a comprehensive understanding of the underlying mechanisms and extent of PH in complex cardiovascular conditions. Advanced CT imaging offers a powerful tool to assess and characterize PH more precisely. CT provides detailed views of pulmonary and aortic anatomy, as well as surrounding vasculature, enabling accurate measurement of pulmonary artery dimensions, which significantly contribute to understanding PH severity [8]. Moreover, CT evaluates the structural relationship between pulmonary vasculature and the aorta, which plays a significant role in predicting outcomes in patients with PH [9, 10].

Emerging evidence suggests that sex differences influence both the severity of PH and the response to TAVI. For instance, women with AS and PH may experience different hemodynamic changes and functional outcomes than men, potentially requiring tailored approaches to assessment and treatment [11–14]. By incorporating sex-specific analysis, CT imaging provides valuable insights into the dynamic relationship between pulmonary circulation and aortic valve pathology and how these differences influence long-term prognosis.

This manuscript aims to bridge the gap between TAVI, PH, advanced imaging, and sex by exploring how CT-based measurements inform risk stratification and mortality prediction. We examine how specific CT parameters—such as pulmonary artery sizes and their relationship with aortic dimensions—refine our understanding of TAVI outcomes across sexes. By leveraging the power of CT imaging, we consider sex-specific factors that go beyond standard pre-TAVI imaging (aortic valve anatomy, vascular access) to improve patient selection and optimize outcomes for male and female TAVI recipients.

## Material and methods

### Study population

A retrospective single-center analysis was conducted on data from TAVI patients at Paracelsus Medical University Hospital, Salzburg, between January 2016 and June 2022. Patients were excluded if they had a bicuspid aortic valve, ascending aortic aneurysm > 50 mm, acute cardiac decompensation at the time of either transthoracic echocardiography (TTE) or TAVI, or a history suggestive of pre-capillary PH (e.g., chronic thromboembolic PH, idiopathic PH, interstitial lung disease, or rheumatologic conditions with pulmonary involvement).

### Ethics declaration

The ethics committee of the state of Salzburg, Austria (EK-Nr. 1082/2024) granted approval for this study. Data management complied with the ethical standards outlined in the Declaration of Helsinki and the International Council for Harmonization—Good Clinical Practice guidelines. The retrospective design allowed the State of Salzburg Ethics Commission to waive the requirement for written informed consent.

### Data extraction

Information was sourced from the ORBIS electronic medical records platform (Agfa Healthcare, Version 08043301.04110DACHL) and the medical archiving system (Softworx by Andreas Schwab TM, 2008). The dataset encompassed patient files, admission/discharge records, and reports from echocardiography and imaging conducted during hospitalization for the TAVI procedure.

### Transthoracic echocardiography

TTE was routinely carried out approximately 1–2 months prior to the TAVI procedure using either an iE33 or Epiq 5 ultrasound system (Philips Healthcare). The assessments were performed by skilled clinicians with more than four years of specialized training in echocardiography. Current valid European Society of Cardiology (ESC) guidelines were applied to classify AS. Left ventricular ejection fraction (LVEF) was examined using Simpson’s method. Systolic pulmonary artery pressure (sPAP) was calculated from pulmonary artery pressure (PAP) derived from maximum tricuspid regurgitant jet velocity (TRVmax) by the simplified Bernoulli equation and right atrial pressure (RAP). TRVmax was measured via continuous wave Doppler over the tricuspid valve, while RAP was estimated by assessing the inferior vena cava (IVC) diameter, which reflects central venous pressure. Further details on RAP estimation were provided recently [15]. The simplified Bernoulli equation was employed to calculate sPAP.

PH was defined based on three key characteristics, in accordance with the ESC guidelines [16]: sPAP (= PAP + RAP) ≥ 40 mmHg, TRVmax ≥ 2.8 m/s, and Tricuspid Annular Plane Systolic Excursion (TAPSE)/sPAP ratio < 0.55 mm/mmHg.

### Computed tomography angiography and measurement of pulmonary and aortic anatomy

CT angiography was performed after echocardiographic confirmation of severe AS as part of the standardized pre-procedural TAVI work-up, typically during the same diagnostic hospital admission within the 1–2 months preceding the intervention and prior to final procedural planning [17, 18]. Participants underwent Electrocardiogram-gated non-contrast CT of the heart and CTA of the aorta pre-procedurally. Imaging assessed the aortic annulus, valve calcium score, vessel anatomy, and vascular access. Scans were conducted using second-generation, multi-detector 256- or 128-slice dual-source (General Electric or Siemens Healthcare). Tube voltage (80–120 kVp) and current modulation are adjusted based on patient size. Two experienced investigators, blinded to all clinical and hemodynamic data, conducted the following measurements in the mediastinal window setting on axial vessel cross-sections of double oblique CT angiographic images, as recommended in previous studies:

1. The largest short-axis diameter of the main pulmonary artery (MPA) is within 3 cm of the pulmonary trunk bifurcation.
2. The largest short-axis diameter of the ascending aorta (AA) at the level of the pulmonary trunk bifurcation.
3. The largest short-axis diameter of the right pulmonary artery (RPA).
4. The largest short-axis diameter of the left pulmonary artery (LPA).

Using these measurements, the ratios of MPA/AA, RPA/AA, and LPA/AA were calculated. Additionally, the diameters of MPA, RPA, and LPA were indexed to body surface area (BSA) using the DuBois formula: BSA = 0.007184 × Height0.725 × Weight0.425. All analyses were performed on stationary workstations (Impax, Agfa-Gevaert).

### TAVI procedure

All patients underwent transfemoral TAVI using second- or third-generation valve systems (CoreValve™ Evolut™ R/Pro). Pre-procedural imaging (TTE, CTA, TEE) facilitated diagnosis and procedure planning, including valve size selection.

### Outcomes

The study’s outcome was overall long-term survival, monitored from the TAVI procedure date to a maximum follow-up of 107 months. Although the maximum individual follow-up extended to 107 months, Kaplan–Meier curves were truncated at 84 months to ensure adequate numbers at risk and avoid unstable survival estimates at very late time points. All-cause mortality was used as a robust endpoint given the lack of consistent cause-specific mortality data and the high burden of competing risks in this elderly population.

### Statistical analysis

Statistical analyses were conducted using SPSS Statistics (v25) and R (v4.2.3). Propensity score matching minimized confounding, balancing cohorts by sex and PH criteria. Matching was performed by exact matching for sex, followed by nearest-neighbor matching for sPAP and TRVmax, using a caliper width of 0.2 standard deviations. Post-matching balance was confirmed in sex distribution and PH criteria.

Normality was assessed using the Kolmogorov-Smirnov test and Q-Q plots. Metrics were presented as mean ± SD (normal) or median ± IQR (non-normal). Categorical data were expressed as absolute numbers and percentages. Student’s *t*-tests, Mann–Whitney *U*-tests, and χ² tests were used as appropriate. ROC curve analysis determined cut-offs for PH parameters (e.g., sPAP ≥ 40 mmHg), and area under the receiver operating characteristic (AUROC) values identified sex-specific thresholds. Kaplan–Meier curves and log-rank tests evaluated survival differences. Cox regression identified predictors of overall survival; variables with *p* < 0.100 in univariate analysis were included in multivariable models. Significance was defined as *p* ≤ 0.050.

## Results

### Study cohort

The initial study cohort consisted of 585 patients who underwent TAVI. Of these, 38 were excluded due to missing CT data, and 10 were excluded due to a potential pre-capillary PH history, leaving 537 patients eligible for analysis. After propensity score matching, the final study cohort comprised 526 patients, evenly divided between males (*n* = 263) and females (*n* = 263).

### Baseline characteristics

Table 1 provides an overview of the baseline characteristics. The mean age was 82.1 ± 5.2 years, with 71.5% aged over 80 and no significant sex differences. Females showed slightly higher LVEF (54.2% vs 51.9%, *p* = 0.012), while males had larger Left Ventricular End-Diastolic Diameter (48.7 mm vs 44.3 mm, *p* < 0.001). Hypertension (87.1%) was the most common comorbidity, with no significant sex differences in other conditions. However, females more often presented with advanced New York Heart Association (NYHA) class ≥ III (46.0% vs 34.6%, *p* = 0.012).

**Table 1:** Baseline characteristics of study cohort

	Total	Male	Female	*p*-value
**No. (%)**				
Total	526 (100.0)	263 (100.0)	263 (100.0)	-
Age				
≤ 80	150 (28.5)	82 (31.2)	68 (25.9)	0.176
> 80	376 (71.5)	181 (68.8)	195 (51.9)	0.176
BMI				
< 18.5	14 (2.7)	3 (1.1)	11 (4.2)	0.027
18.5–24.9	236 (44.9)	107 (40.7)	129 (49.0)	0.044
25.0–29.9	185 (35.2)	114 (43.4)	71 (27.0)	< 0.001
30.0–34.9	67 (12.7)	30 (11.4)	37 (14.1)	0.367
35.0–39.9	20 (3.8)	8 (3.0)	12 (4.6)	0.334
≥ 40.0	4 (0.7)	1 (0.4)	3 (1.1)	0.549
NYHA ≥ III	212 (40.3)	91 (34.6)	121 (46.0)	0.012
Diabetes mellitus	144 (27.4)	76 (28.9)	68 (25.9)	0.434
Arterial hypertension	458 (87.1)	226 (85.9)	232 (88.2)	0.426
CHD	265 (50.4)	136 (51.7)	129 (49.0)	0.542
AF	189 (35.9)	94 (35.7)	95 (36.1)	0.928
PAOD	44 (8.4)	22 (8.4)	22 (8.4)	1.000
COPD	60 (11.4)	32 (12.2)	28 (10.6)	0.583
MI—prehistory	37 (7.0)	16 (6.1)	11 (4.2)	0.394
Stroke—prehistory	39 (7.4)	19 (7.2)	20 (7.6)	0.868
Graduation of AS^*^				
High gradient AS	474 (90.1)	234 (89.0)	240 (91.2)	0.381
Low gradient AS	52 (8.9)	29 (11.0)	23 (8.8)	0.381
Mean ± SD				
Age (years)	82.1 ± 5.2	81.8 ± 5.4	82.4 ± 4.9	0.182
Height (cm)	167.8 ± 8.8	174.0 ± 6.0	161.5 ± 6.3	< 0.001
Weight (kg)	72.9 ± 14.7	79.1 ± 12.9	66.7 ± 13.8	< 0.001
BMI (kg/m^2^)	25.8 ± 4.5	26.1 ± 3.8	25.5 ± 5.0	0.130
BSA (m^2^)	1.8 ± 0.2	1.9 ± 0.2	1.7 ± 0.2	< 0.001
LVEF (%)	53.0 ± 9.8	51.9 ± 10.9	54.2 ± 8.4	0.012
LVEDD (mm)	46.4 ± 6.8	48.7 ± 6.5	44.3 ± 6.3	< 0.001
IVSd (mm)	13.4 ± 2.1	13.5 ± 2.2	13.4 ± 2.0	0.449
AV Vmax (m/s)	4.4 ± 0.6	4.3 ± 0.6	4.4 ± 0.5	0.060
AV MPG (mmHg)	45.6 ± 12.3	45.5 ± 12.6	45.8 ± 11.9	0.128
TAPSE (mm)	21.9 ± 4.7	22.0 ± 5.0	21.8 ± 4.5	0.572
Median ± IQR				
Creatinine (mg/dL)	1.1 ± 0.5	1.1 ± 0.5	1.0 ± 0.5	0.172
HK (%)	38.0 ± 6.7	38.3 ± 6.5	37.6 ± 7.4	0.844
HB (g/dL)	12.8 ± 2.1	12.9 ± 2.2	12.7 ± 2.0	0.540
CK (U/L)	81.0 ± 60.0	84.5 ± 66.0	78.0 ± 51.8	0.954
proBNP (pg/mL)	2036.0 ± 3488.5	2116.0 ± 3451.5	1807.0 ± 3572.8	0.530

*BMI* body mass index, *NYHA* New York Heart Association, *CHD* coronary heart disease, *AF* atrial fibrillation, *PAOD* peripheral arterial occlusive disease, *COPD* chronic obstructive pulmonary disease, *MI* myocardial infarction, *AS* aortic stenosis, *BSA* body surface area, *LVEF* left ventricular ejection fraction, *LVEDD* left ventricular enddiastolic diameter, *IVSD* interventricular septal thickness, *AV Vmax* aortic valve maximal systolic transvalvular flow velocity, *AV MPG* aortic valve mean systolic pressure gradient, *TAPSE* tricuspid annular plane systolic excursion, *HK* hematocrit, *HB* hemoglobin, *SD* standard deviation, *IQR* interquartile range

### Echocardiographic and radiological characteristics of PH

Table 2 outlines the cardiological and radiological parameters used to evaluate PH.

**Table 2:** Cardiological and radiological criteria of PH

	Total	Male	Female	*p*-value
Cardiological criteria of PH				
Mean ± SD				
sPAP (mmHg)	38.5 ± 17.0	38.6 ± 18.6	38.4 ± 15.4	0.920
TRVmax (m/s)	2.7 ± 0.8	2.6 ± 0.9	2.7 ± 0.8	0.384
TAPSE/sPAP (mm/mmHg)	0.8 ± 0.8	0.9 ± 0.9	0.7 ± 0.7	0.096
No. (%)				
sPAP ≥ 40 mmHg	231 (43.9)	120 (45.6)	111 (42.2)	0.340
TRVmax ≥ 2.8 m/s	239 (45.4)	122 (46.4)	117 (44.5)	0.546
TAPSE/sPAP < 0.55 mm/mmHg	230 (43.7)	117 (44.5)	113 (43.0)	0.609
Radiological criteria of PH				
Mean ± SD				
MPA (mm)	28.9 ± 5.0	29.6 ± 5.2	28.4 ± 4.8	0.004
MPA/BSA (mm/m^2^)	16.1 ± 3.1	15.4 ± 2.8	16.8 ± 3.1	< 0.001
MPA/AA	0.8 ± 0.2	0.8 ± 0.2	0.8 ± 0.1	0.044
LPA (mm)	25.5 ± 3.3	25.9 ± 3.3	25.0 ± 3.2	0.001
LPA/BSA (mm/m^2^)	14.1 ± 2.1	13.4 ± 1.7	14.8 ± 2.2	< 0.001
LPA/AA	0.7 ± 0.1	0.7 ± 0.1	0.8 ± 0.1	0.004
RPA (mm)	26.8 ± 3.8	27.7 ± 3.9	25.8 ± 3.5	< 0.001
RPA/BSA (mm/m^2^)	14.8 ± 2.4	14.4 ± 2.1	15.3 ± 2.6	< 0.001
RPA/AA	0.8 ± 0.1	0.8 ± 0.1	0.8 ± 0.1	0.736
AA (mm)	35.1 ± 4.2	36.4 ± 4.1	33.8 ± 4.0	< 0.001

*sPAP* systolic pulmonary artery pressure, *TRVmax* maximum tricuspid regurgitant jet velocity, *TAPSE* tricuspid annular plane systolic excursion, *MPA* main pulmonary artery, *LPA* left pulmonary artery, *RPA* right pulmonary artery, *BSA* body surface area, *AA* ascending aorta

#### Cardiological criteria

The mean sPAP was 38.5 ± 17.0 mmHg, with no significant difference between males (38.6 ± 18.6 mmHg) and females (38.4 ± 15.4 mmHg, *p* = 0.920). Similarly, TRVmax averaged 2.7 ± 0.8 m/s with no sex-specific differences (*p* = 0.384). The TAPSE/sPAP ratio, used as a functional marker of right ventricular efficiency, was 0.8 ± 0.8 mm/mmHg and trended lower in females (0.7 ± 0.7 mm/mmHg) than males (0.9 ± 0.9 mm/mmHg, *p* = 0.096). Elevated PH markers were common: 43.9% had sPAP ≥ 40 mmHg, 45.4% had TRVmax ≥ 2.8 m/s, and 43.7% had TAPSE/sPAP < 0.55 mm/mmHg, with no significant sex differences.

#### Radiological criteria

Radiological markers revealed significant sex-specific variations. The mean MPA diameter was larger in males (29.6 ± 5.2 mm) than in females (28.4 ± 4.8 mm, *p* = 0.004), while MPA indexed to BSA (MPA/BSA) was greater in females (16.8 ± 3.1 mm/m²) than in males (15.4 ± 2.8 mm/m², *p* < 0.001). LPA and RPA diameters were larger in males, but indexed values were consistently higher in females (*p* < 0.001). The AA diameter also differed between sexes (male vs female: 36.4 ± 4.1 mm vs 33.8 ± 4.0 mm, *p* < 0.001). Ratios such as MPA/AA or MPA/BSA were significantly higher in females, reflecting relative differences in vascular dimensions.

### AUROC analysis

**Fig. 1:**
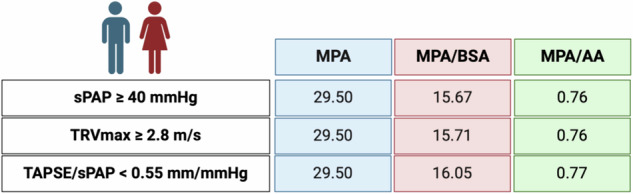
Overview of non-sex-specific cut-off values of MPA metrics in PH detection. AA, ascending aorta; BSA, body surface area; MPA, main pulmonar artery; sPAP, systolic pulmonary artery pressure; TAPSE, tricuspid annular plane systolic excursion; TRVmax, maximum tricuspid regurgitant jet velocity

**Fig. 2:**
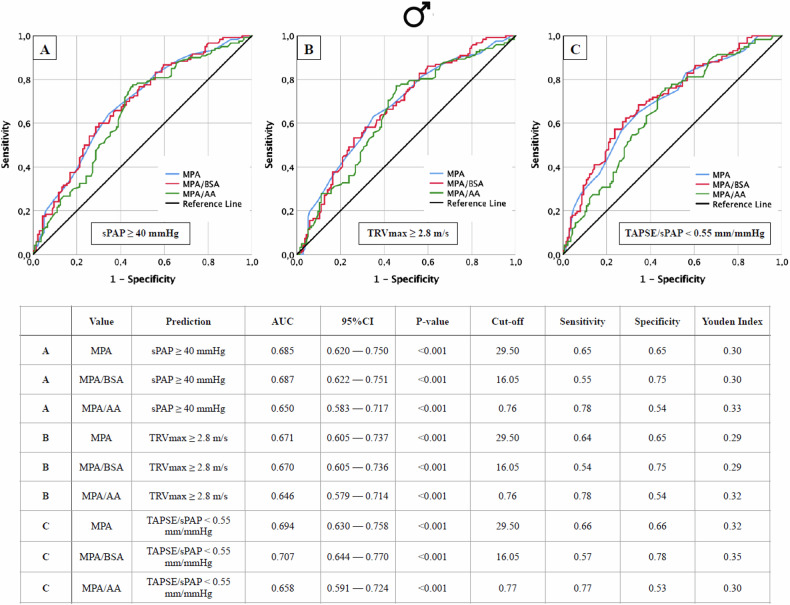
AUROC analyses of MPA, MPA/BSA, and MPA/AA for the prediction of different echocardiographic criteria of PH, with concerning cut-off values, Youden Index, sensitivity, and specificity in dependence on male sex. Echocardiographic criteria: **A** sPAP ≥ 40 mmHg; **B** TRVmax ≥ 2.8 m/s; **C** TAPSE/sPAP < 0.55 mm/mmHg. AA, ascending aorta; AUC, area under the curve; BSA, body surface area; MPA, main pulmonar artery; sPAP, systolic pulmonary artery pressure; TAPSE, tricuspid annular plane systolic excursion; TRVmax, maximum tricuspid regurgitant jet velocity

**Fig. 3:**
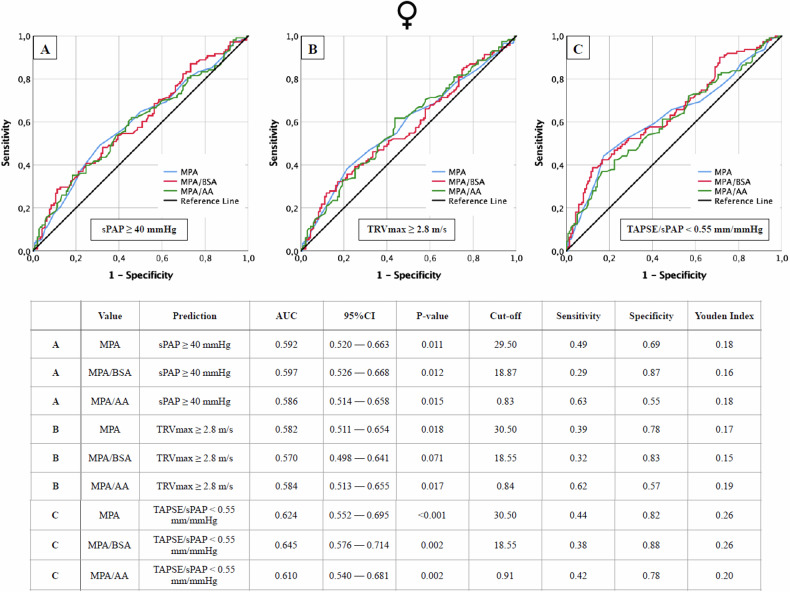
AUROC analyses of MPA, MPA/BSA, and MPA/AA for the prediction of different echocardiographic criteria of PH, with concerning cut-off values, Youden Index, sensitivity, and specificity in dependence on female sex. Echocardiographic criteria: **A** sPAP ≥ 40 mmHg; **B** TRVmax ≥ 2.8 m/s; **C** TAPSE/sPAP < 0.55 mm/mmHg. AA, ascending aorta; AUC, area under the curve; BSA, body surface area; MPA, main pulmonar artery; sPAP, systolic pulmonary artery pressure; TAPSE, tricuspid annular plane systolic excursion; TRVmax, maximum tricuspid regurgitant jet velocity

**Fig. 4:**
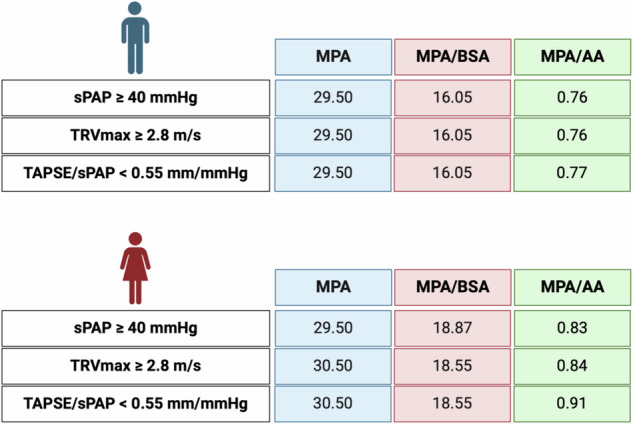
Overview of sex-specific cut-off values of MPA metrics in PH detection. AA, ascending aorta; BSA, body surface area; MPA, main pulmonary artery; sPAP, systolic pulmonary artery pressure; TAPSE, tricuspid annular plane systolic excursion; TRVmax, maximum tricuspid regurgitant jet velocity

To establish radiological cut-off values for PH, echocardiographic parameters (sPAP ≥ 40 mmHg, TRVmax ≥ 2.8 m/s, TAPSE/sPAP < 0.55 mm/mmHg) were analyzed using AUROC. Results, including sensitivity, specificity, and Youden-Index, are presented for the overall cohort in Supplementary Figs. 1–3, and for sex-specific differences in Figs. 1 and 2. Figure 3 (non-sex-specific) and Fig. 4 (sex-specific) provide clear graphical representations of the cut-off values obtained.

#### Non-sex-specific cut-off values for the CT parameter to predict PH

MPA and its ratios (MPA/BSA and MPA/AA) outperformed LPA and RPA in predicting PH. Non-sex-specific cut-offs (Fig. 3) were: MPA = 29.50 mm, MPA/BSA = 15.67–16.05 mm/m², and MPA/AA = 0.76–0.77. Supplementary Figs. 1–3 confirmed the weaker predictive performance of LPA and RPA metrics. MPA and its indexed ratios achieved the highest diagnostic utility and were selected for sex-specific analysis.

#### Sex-specific cut-off values for the CT parameter to predict PH

Sex-specific cut-offs for MPA and its ratios (Fig. 1: male; Fig. 2: female) revealed that male patients consistently had higher AUROC values (AUC 0.646–0.707; *p* < 0.001) compared to females (AUC 0.570–0.645; *p* = 0.001–0.071). Cut-off values for males were nearly identical to non-sex-specific values, while females showed significant deviations in indexed parameters such as MPA/BSA and MPA/AA.

### Kaplan–Meier curves for survival post TAVI depending on CT parameters

In the overall cohort, median survival after TAVI was 81.9 months (95% confidence interval (CI): 78.9–85.0), as estimated by Kaplan–Meier analysis accounting for censoring. Kaplan–Meier–estimated survival rates were 91.6% at 12 months, 76.8% at 36 months, and 46.6% at 60 months.

**Fig. 5:**
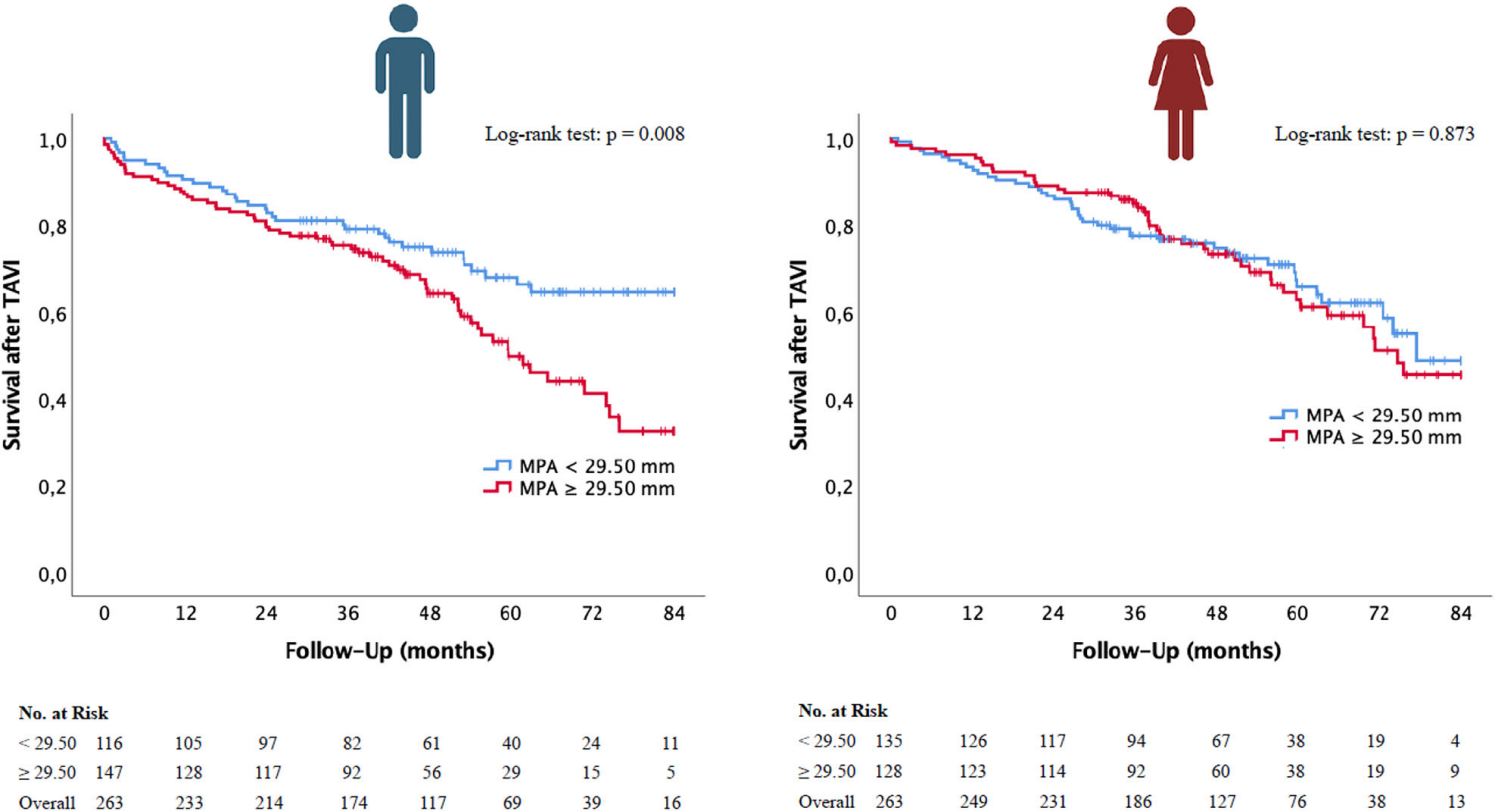
Kaplan–Meier curve with corresponding numbers at risk and log-rank tests for the detection of overall mortality in dependence on MPA cut-off values. MPA, main pulmonary artery diameter; TAVI, transcatheter aortic valve implantation

**Fig. 6:**
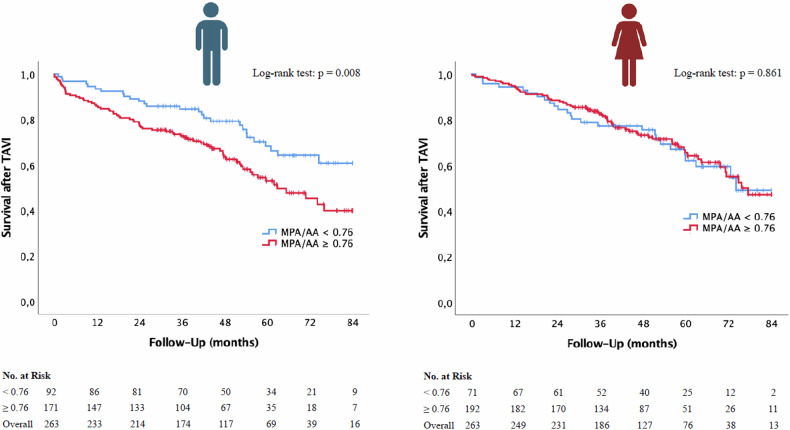
Kaplan–Meier curve with corresponding numbers at risk and log-rank tests for the detection of overall mortality in dependence on MPA/AA cut-off values. AA, ascending aorta; MPA, main pulmonary artery diameter; TAVI, transcatheter aortic valve implantation

Kaplan–Meier curves were constructed to evaluate the significance of non-sex-specific/male-specific radiological cut-off values for PH in relation to overall survival in TAVI patients. The results for MPA, MPA/BSA, and MPA/AA, each stratified by sex, are illustrated in Figs. 5 and 6 and Supplementary Fig. 4.

#### MPA

Figure 5 compares survival between patients with MPA values above and below the cut-off. Male patients with MPA ≥ 29.50 mm had significantly reduced survival (log-rank *p* = 0.008), while differences among females were not statistically significant (log-rank *p* = 0.873).

#### MPA/BSA

Furthermore, overall survival after TAVI based on the MPA/BSA cut-off value showed that male patients with MPA/BSA values ≥ 16.05 mm/m² demonstrated significantly higher mortality at the 84-month follow-up compared to those below this threshold (*p* = 0.038). However, among females with the same cut-off value, no significant differences in survival were observed between the subgroups (*p* = 0.806) (Supplementary Fig. 4).

#### MPA/AA

Finally, Fig. 6 evaluates the MPA/AA cut-off value of ≥ 0.76. Male TAVI patients with MPA/AA values ≥ 0.76 showed significantly poorer long-term survival (*p* = 0.008). Conversely, no significant survival differences were found in the female cohort (*p* = 0.861).

### Cox regression analysis

Cox proportional hazards regression analysis was conducted to evaluate the association between overall survival and echocardiographic/radiological parameters of PH in TAVI patients (Table 3).

**Table 3:** Cox regression analysis

Cox hazard regression	Univariate		Multivariable	
	Hazard ratio (95% CI)	*p*-value	Hazard ratio (95% CI)	*p*-value
Male				
sPAP ≥ 40 mmHg	1.466 (1.014–2.120)	0.041	0.849 (0.443–1.625)	0.621
TRVmax ≥ 2.8 m/s	1.189 (0.824–1.715)	0.356		
TAPSE/sPAP < 0.55 mm/mmHg	1.555 (1.076–2.248)	0.019	1.385 (0.917–2.094)	0.122
MPA ≥ 29.50 mm	1.743 (1.152–2.637)	0.009	1.294 (0.750–2.235)	0.354
MPA/BSA ≥ 16.05 mm/m^2^	1.516 (1.020–2.254)	0.040	1.032 (0.626–1.702)	0.900
MPA/AA ≥ 0.76	1.815 (1.164–2.831)	0.009	1.857 (1.190–2.898)	0.006
Female				
sPAP ≥ 40 mmHg	1.032 (0.697–1.530)	0.874		
TRVmax ≥ 2.8 m/s	1.220 (0.823–1.806)	0.322		
TAPSE/sPAP < 0.55 mm/mmHg	1.096 (0.739–1.623)	0.649		
MPA ≥ 29.50 mm	1.035 (0.681–1.572)	0.873		
MPA/BSA ≥ 16.05 mm/m^2^	1.055 (0.689–1.614)	0.806		
MPA/AA ≥ 0.76	0.960 (0.607–1.519)	0.861		

*sPAP* systolic pulmonary artery pressure, *TRVmax* maximum tricuspid regurgitant jet velocity, *TAPSE* tricuspid annular plane systolic excursion, *MPA* main pulmonary artery, *BSA* body surface area, *AA* ascending aorta

In the univariate analysis for males, several variables showed significant associations with the hazard of the event. Notably, sPAP ≥ 40 mmHg (hazard ratio (HR) = 1.466, *p* = 0.041), TAPSE/sPAP < 0.55 mm/mmHg (HR = 1.555, *p* = 0.019), MPA ≥ 29.50 mm (HR = 1.743, *p* = 0.009), MPA/BSA ≥ 16.05 mm/m² (HR = 1.516, *p* = 0.040), and MPA/AA ≥ 0.76 (HR = 1.815, *p* = 0.009) were significant. However, in the multivariable analysis, only MPA/AA ≥ 0.76 remained significantly associated with the outcome (HR = 1.857, *p* = 0.006), while other variables, such as sPAP and MPA, lost significance.

For females, the univariate analysis of the respective parameters already showed no relevant difference, so the multivariable analysis was omitted.

## Discussion

### Superiority of MPA metrics in PH detection among TAVI patients

MPA and its derived ratios have proven to be more reliable for detecting PH in TAVI patients compared to RPA, LPA, or their respective ratios. This superiority is based on the anatomical and physiological features of MPA.

As the central conduit in pulmonary circulation, the MPA channels the entire right ventricular cardiac output and directly reflects elevated pulmonary pressures—a hallmark of PH—with minimal delay or dampening [19]. In contrast, the RPA and LPA, branching off the MPA, are influenced by factors such as asymmetry in blood flow distribution and regional lung pathologies like atelectasis or hyperinflation [20, 21].

The MPA’s relatively uniform anatomy across individuals enhances its diagnostic reliability compared to the RPA and LPA, which are more prone to variability due to differences in thoracic anatomy, lung volume, and surrounding structures [22]. For instance, the RPA’s proximity to the AA and the LPA’s interaction with the left atrium introduce subtle anatomical distortions that complicate measurements.

In radiological imaging, MPA’s central and fixed location minimizes artifacts caused by respiration or posture, allowing for reproducible assessment [23]. These technical advantages make MPA-derived metrics, such as MPA/AA, inherently more robust for standardized evaluations than RPA or LPA-based measurements.

### Sex-specific discrepancies in PH diagnosis: reevaluating the applicability of current thresholds for men and women

Current ESC guidelines for PH diagnosis have refined thresholds over time, transitioning from an MPA ≥ 29 mm in 2015 [24] to an MPA ≥ 30 mm in 2022 [16]. Despite these refinements, our findings underscore the importance of considering sex-specific anatomical and physiological differences, which remain overlooked in current guidelines.

Men and women exhibit significant variations in thoracic dimensions and vascular diameters even in the absence of pathology [25, 26]. Our data revealed that elevated MPA and MPA/AA ratios were more strongly associated with echocardiographically defined PH in men than women. Men’s larger baseline vascular dimensions and higher compliance likely amplify proportional increases in MPA size under pathological conditions, enhancing the diagnostic sensitivity of these metrics [27].

In women, physiological factors like reduced vascular compliance and smaller baseline pulmonary artery dimensions may limit the sensitivity and specificity of these thresholds, particularly in older age groups where arterial stiffness further compromises diagnostic accuracy [28, 29]. This could lead to underestimation of PH severity or delayed diagnosis when using generalized thresholds.

The relatively lower AUROC values for women in our study suggest that current methodologies may not fully capture the hemodynamic nuances in female TAVI patients. These findings highlight the need for further research to refine sex-specific cut-offs and explore alternative diagnostic tools or biomarkers to enhance accuracy in women.

### Sex-specific dynamics in PH progression, clinical presentation, and survival following TAVI

Our analysis revealed that MPA and, more prominently, the MPA/AA ratio were predictive of survival following TAVI [15] exclusively in male patients. This observation underscores potential sex-specific pathophysiological mechanisms and raises critical questions about PH progression in men undergoing TAVI.

One plausible explanation could be the more indolent clinical progression of PH in men with severe AS [30], which may delay recognition and intervention. Male patients in our cohort frequently presented with a lower NYHA functional class, reflecting milder reported symptoms despite significant hemodynamic burden. Remarkably, males also exhibited slightly reduced LVEF compared to females, possibly contributing to PH pathogenesis. As the left ventricle struggles to sustain the pressure overload imposed by severe AS, its contractile function deteriorates, resulting in volume overload and retrograde pressure transmission into the pulmonary circulation. This pulmonary congestion exacerbates PH, driving its progression to clinically significant levels [31, 32]. The interplay between ventricular dysfunction and worsening pulmonary pressures in men highlights how left ventricular “breakdown” function acts as a pivotal event that amplifies the clinical impact of PH. Consequently, the late-stage clinical presentation of PH in men not only reflects delayed detection but also highlights a pathophysiological cascade characterized by compounded hemodynamic burdens.

These findings suggest that men may tolerate higher pulmonary pressures without a proportional increase in symptoms, delaying clinical attention. Heightened clinical vigilance in male TAVI candidates, particularly those with subtle or non-specific symptoms, is critical. Sensitive screening strategies, such as systematic imaging of the MPA/AA ratio or complementary biomarkers, could facilitate earlier PH detection in men, potentially improving PH-related risk stratification. In contrast, the limited prognostic relevance of the MPA/AA ratio in female TAVI patients likely reflects sex-specific differences in PH phenotype. Women with severe AS more frequently exhibit post-capillary PH related to Heart Failure with preserved Ejection Fraction, which is associated with less pronounced pulmonary vascular remodeling and preserved right ventricular-pulmonary arterial coupling [11–14]. Under these conditions, static CT-derived vascular dimensions may fail to capture prognostically meaningful disease severity. Smaller baseline thoracic and aortic dimensions together with age-related increases in arterial stiffness may further constrain the discriminatory capacity of MPA-based metrics in women [25, 26, 28, 29].

### Limitation

This study has several limitations. Its retrospective, single-center design introduces potential selection bias and limits causal inference and generalizability. PH was assessed using echocardiographic criteria, as invasive hemodynamic confirmation by right heart catheterization—the diagnostic gold standard—was not available. Consequently, the reliable exclusion of pre-capillary or combined forms of PH was limited, and despite exclusion based on clinical history, residual misclassification of PH etiology cannot be ruled out. Given that pre-capillary PH may be associated with distinct patterns of pulmonary artery remodeling, this may have influenced the observed associations.

Although propensity score matching was applied to reduce confounding, unmeasured factors such as frailty and comorbidities may still have affected outcomes. In addition, cause-specific mortality data were not available; therefore, survival analyses reflect all-cause mortality and may be influenced by non-cardiovascular causes of death, which is particularly relevant in this elderly TAVI population with substantial competing risks and limited post-procedural life expectancy. Finally, multivariable models were adjusted for PH–related parameters; thus, the observed associations indicate independence from other PH metrics rather than full prognostic independence. Future multicenter, prospective studies are warranted to address these limitations.

## Conclusion

In conclusion, this study underscores the value of CT imaging as a complementary tool for the assessment of PH in TAVI patients and demonstrates pronounced sex-specific differences in pulmonary artery dimensions. In male patients, the MPA/AA ratio was associated with survival after TAVI, independent of other PH-related imaging and echocardiographic parameters. These findings suggest that CT-derived pulmonary artery metrics may provide additional prognostic information within the context of PH-related risk stratification rather than serving as comprehensive predictors of post-TAVI survival. Future studies should aim to validate these observations in larger, multicenter cohorts, further elucidate the mechanisms underlying sex-specific differences, and explore the integration of complementary imaging modalities. Such efforts may ultimately support more individualized risk assessment strategies in TAVI patients.

## Supplementary information

ELECTRONIC SUPPLEMENTARY MATERIAL

## Data Availability

The data underlying this article will be shared upon reasonable request to the corresponding author.

## Abbreviations

AA: Ascending aorta
AUC: Area under the curve
BSA: Body surface area
LPA: Left pulmonary artery
MPA: Main pulmonary artery
RPA: Right pulmonary artery
sPAP: Systolic pulmonary artery pressure
TAPSE: Tricuspid annular plane systolic excursion
TAVI: Transcatheter aortic valve implantation
TRVmax: Maximum tricuspid regurgitant jet velocity
