# Supplementary material for: Sex-specific utility of pulmonary artery metrics in predicting pulmonary hypertension and survival after TAVI: insights from advanced CT imaging
Source: Insights Imaging. 2026 Apr 7;17:91. doi: 10.1186/s13244-026-02272-x (PMC13057069; doi:10.1186/s13244-026-02272-x)
Supplement: Supplementary file 1 — ELECTRONIC SUPPLEMENTARY MATERIAL [file 13244_2026_2272_MOESM1_ESM.pdf]

Sex-Specific Utility of Pulmonary Artery Metrics in Predicting Pulmonary Hypertension and Survival After TAVI: Insights from Advanced CT Imaging

ELECTRONIC SUPPLEMENTARY MATERIAL

Supplementary Figure 1
AUROC analyses of different radiological parameters for prediction of sPAP ≥ 40 mmHg with concerning cut-off values, Youden Index, sensitivity and specificity of overall study cohort

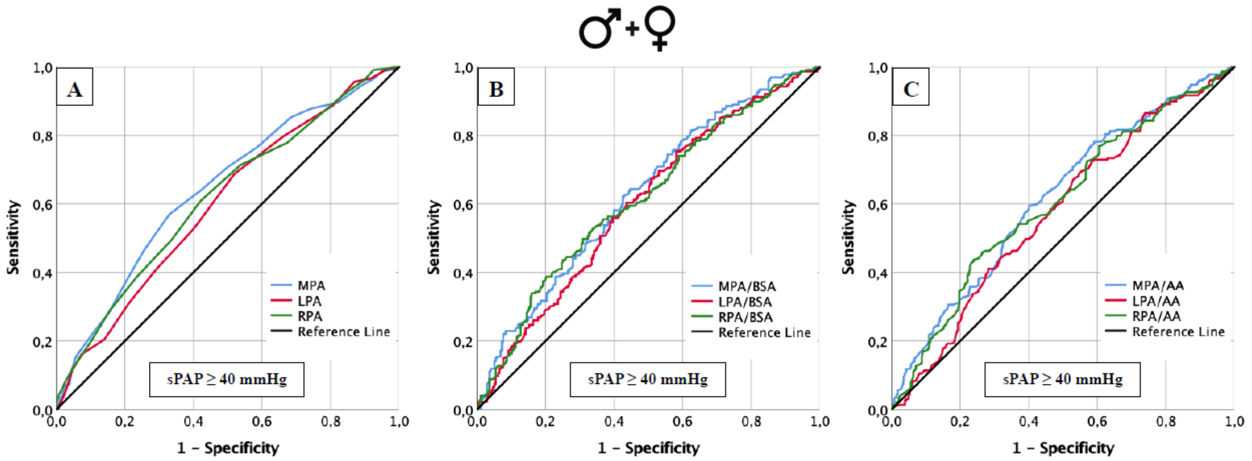

	Value	Prediction	AUC	95%CI	P-value	Cut-off	Sensitivity	Specificity	Youden Index
A	MPA	sPAP ≥ 40 mmHg	0.644	0.596 — 0.691	<0.001	29.50	0.57	0.67	0.24
A	LPA	sPAP ≥ 40 mmHg	0.601	0.553 — 0.650	<0.001	24.50	0.69	0.48	0.17
A	RPA	sPAP ≥ 40 mmHg	0.619	0.570 — 0.667	<0.001	26.50	0.61	0.58	0.19
B	MPA/BSA	sPAP ≥ 40 mmHg	0.629	0.581 — 0.678	<0.001	15.67	0.63	0.57	0.20
B	LPA/BSA	sPAP ≥ 40 mmHg	0.600	0.550 — 0.649	<0.001	13.50	0.75	0.42	0.17
B	RPA/BSA	sPAP ≥ 40 mmHg	0.615	0.566 — 0.664	<0.001	15.07	0.52	0.67	0.19
C	MPA/AA	sPAP ≥ 40 mmHg	0.618	0.570 — 0.666	<0.001	0.76	0.78	0.41	0.19
C	LPA/AA	sPAP ≥ 40 mmHg	0.574	0.525 — 0.624	0.004	0.69	0.73	0.41	0.14
C	RPA/AA	sPAP ≥ 40 mmHg	0.603	0.554 — 0.652	<0.001	0.81	0.44	0.76	0.20

MPA: main pulmonary artery diameter; LPA: left pulmonary artery diameter; RPA: right pulmonary artery diameter; BSA: body surface area; AA: ascending aorta; AUC: area under the curve; sPAP: systolic pulmonary artery pressure

Supplementary Figure 2
AUROC analyses of different radiological parameters for prediction of TRVmax ≥ 2.8 m/s with concerning cut-off values, Youden Index, sensitivity and specificity of overall study cohort

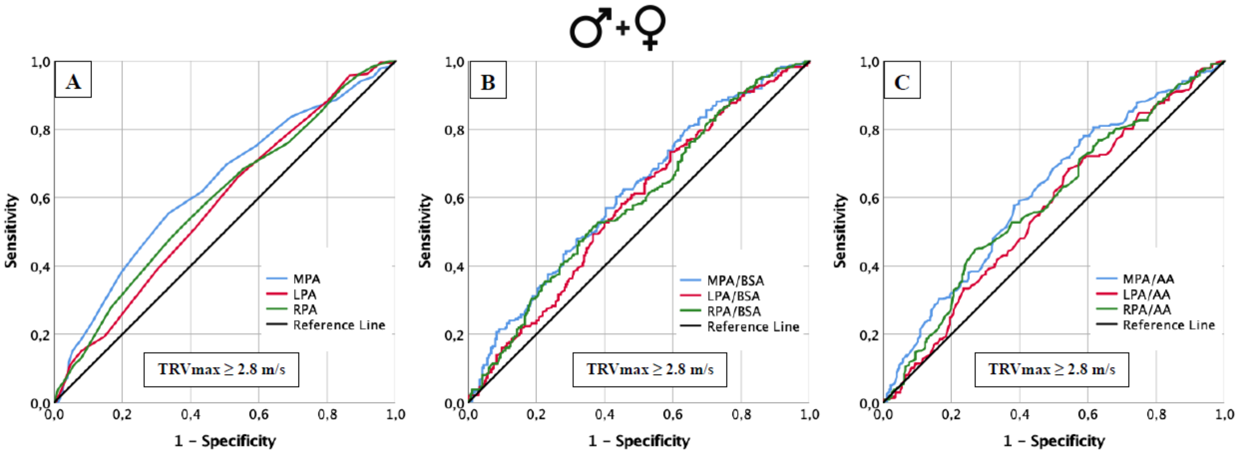

	Value	Prediction	AUC	95%CI	P-value	Cut-off	Sensitivity	Specificity	Youden Index
A	MPA	TRVmax ≥ 2.8 m/s	0.632	0.584 — 0.680	<0.001	29.50	0.56	0.66	0.22
A	LPA	TRVmax ≥ 2.8 m/s	0.580	0.531 — 0.629	0.002	24.50	0.66	0.46	0.12
A	RPA	TRVmax ≥ 2.8 m/s	0.594	0.545 — 0.643	<0.001	26.50	0.58	0.56	0.14
B	MPA/BSA	TRVmax ≥ 2.8 m/s	0.613	0.564 — 0.662	<0.001	15.71	0.60	0.57	0.17
B	LPA/BSA	TRVmax ≥ 2.8 m/s	0.577	0.527 — 0.627	0.003	13.15	0.73	0.41	0.14
B	RPA/BSA	TRVmax ≥ 2.8 m/s	0.586	0.536 — 0.636	0.001	15.04	0.50	0.65	0.15
C	MPA/AA	TRVmax ≥ 2.8 m/s	0.617	0.568 — 0.665	<0.001	0.76	0.78	0.41	0.19
C	LPA/AA	TRVmax ≥ 2.8 m/s	0.563	0.514 — 0.613	0.013	0.70	0.68	0.46	0.14
C	RPA/AA	TRVmax ≥ 2.8 m/s	0.587	0.538 — 0.636	0.001	0.80	0.45	0.73	0.18

MPA: main pulmonary artery diameter; LPA: left pulmonary artery diameter; RPA: right pulmonary artery diameter; BSA: body surface area; AA: ascending aorta; AUC: area under the curve; TRVmax: maximum tricuspid regurgitant jet velocity

Supplementary Figure 3
AUROC analyses of different radiological parameters for prediction of TAPSE/sPAP < 0.55 mm/mmHg with concerning cut-off values, Youden Index, sensitivity and specificity of overall study cohort

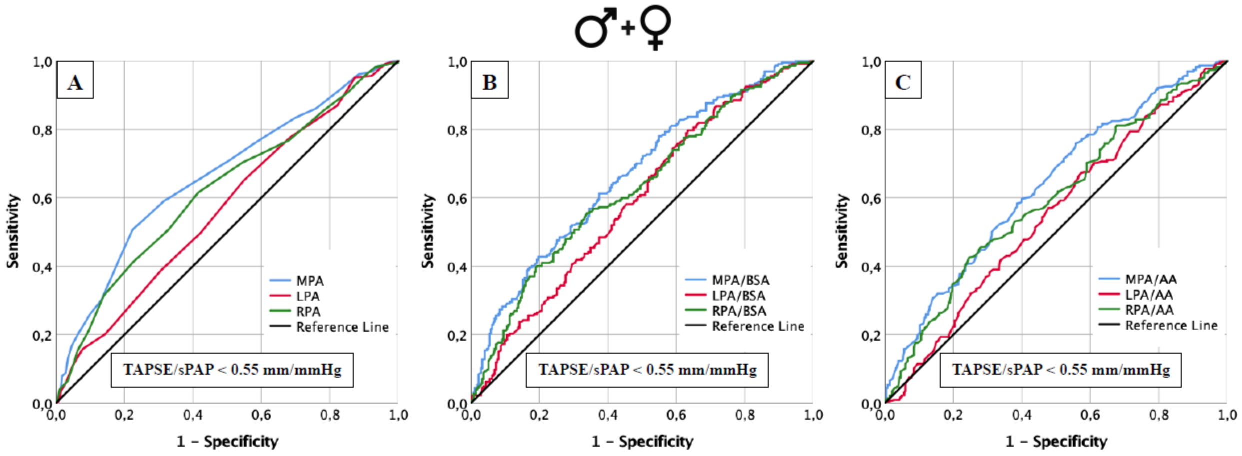

	Value	Prediction	AUC	95%CI	P-value	Cut-off	Sensitivity	Specificity	Youden Index
A	MPA	TAPSE/sPAP < 0.55 mm/mmHg	0.663	0.616 — 0.710	<0.001	29.50	0.59	0.69	0.28
A	LPA	TAPSE/sPAP < 0.55 mm/mmHg	0.570	0.520 — 0.619	0.006	24.50	0.65	0.45	0.10
A	RPA	TAPSE/sPAP < 0.55 mm/mmHg	0.621	0.572 — 0.670	<0.001	26.50	0.62	0.58	0.20
B	MPA/BSA	TAPSE/sPAP < 0.55 mm/mmHg	0.666	0.619 — 0.713	<0.001	16.05	0.61	0.63	0.24
B	LPA/BSA	TAPSE/sPAP < 0.55 mm/mmHg	0.593	0.544 — 0.643	<0.001	13.15	0.74	0.41	0.16
B	RPA/BSA	TAPSE/sPAP < 0.55 mm/mmHg	0.629	0.580 — 0.677	<0.001	14.97	0.56	0.66	0.22
C	MPA/AA	TAPSE/sPAP < 0.55 mm/mmHg	0.635	0.587 — 0.683	<0.001	0.77	0.76	0.44	0.20
C	LPA/AA	TAPSE/sPAP < 0.55 mm/mmHg	0.549	0.499 — 0.599	0.056	0.70	0.67	0.43	0.10
C	RPA/AA	TAPSE/sPAP < 0.55 mm/mmHg	0.594	0.545 — 0.644	<0.001	0.80	0.46	0.72	0.18

MPA: main pulmonary artery diameter; LPA: left pulmonary artery diameter; RPA: right pulmonary artery diameter; BSA: body surface area; AA: ascending aorta; AUC: area under the curve; TAPSE: tricuspid annular plane systolic excursion; sPAP: systolic pulmonary artery pressure

Supplementary Figure 4

Kaplan-Meier curve with corresponding numbers at risk and log-rank tests for detection of overall mortality in dependence MPA/BSA cut-off values

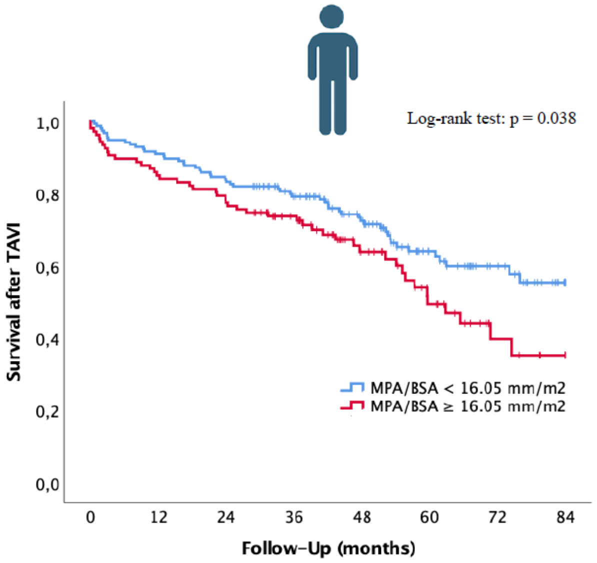

No. at Risk								
< 16.05	156	142	131	110	80	47	30	11
≥ 16.05	107	91	83	64	37	22	9	5
Overall	263	233	214	174	117	69	39	16

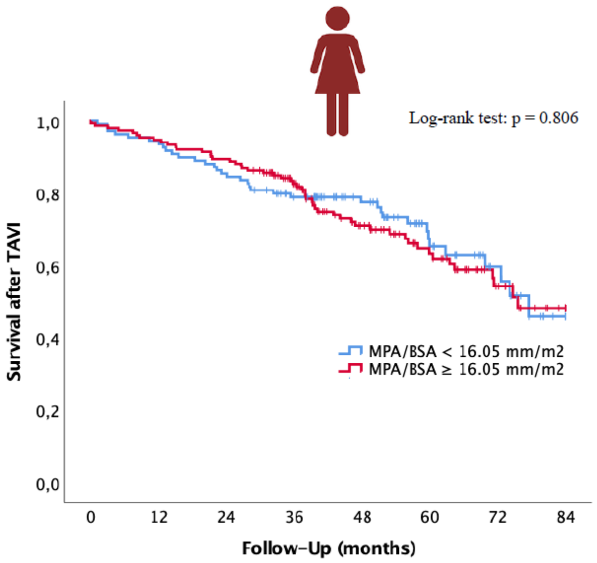

No. at Risk								
< 16.05	110	104	94	79	58	31	15	3
≥ 16.05	153	145	137	107	69	45	23	10
Overall	263	249	231	186	127	76	38	13

TAVI: transcatheter aortic valve implantation; MPA: main pulmonary artery diameter; BSA: body surface area
